# Left ventricular hypertrophy: an ECG-based study of prevalence and risk factors in a multiethnic population

**DOI:** 10.1136/openhrt-2023-002495

**Published:** 2023-11-07

**Authors:** Hina Taki, Jaakko Tuomilehto, Paul Zimmet, Abdonas Tamosiunas, Sudhir Kowlessur, Dianna J Magliano, Jonathan E Shaw, Stefan Söderberg, Ulf Nilsson

**Affiliations:** 1 Department of Public Health and Clinical Medicine, Umeå University, Umeå, Sweden; 2 Public Health Unit, Finnish Institute for Health and Welfare, Helsinki, Finland; 3 Department of Public Health, University of Helsinki, Helsinki, Finland; 4 Department of Diabetes, Central Clinical School, Monash University, Melbourne, Victoria, Australia; 5 Institute of Cardiology, Lithuanian University of Health Sciences, Kaunas, Lithuania; 6 Ministry of Health and Wellness, Port Louis, Mauritius; 7 Diabetes and Population Health, Baker Heart and Diabetes Institute, Melbourne, Victoria, Australia

**Keywords:** Electrocardiography, Hypertension, Diabetes Mellitus, EPIDEMIOLOGY

## Abstract

**Background:**

Left ventricular hypertrophy (LVH) is frequently seen in association with arterial hypertension and indicates poor prognosis. This study aimed to determine the prevalence of LVH and associated factors in a multiethnic population from Mauritius.

**Methods:**

Population-based health surveys were performed in 2009 and 2015 and included in total 8961 individuals aged 35–75 years with recorded 12-lead ECG. LVH was defined according to three criteria: Sokolow-Lyon, Cornell voltage and Cornell product. Data were collected about health and lifestyle behaviour. Anthropometry and blood pressure were measured. Fasting levels of blood lipids and glucose were determined, oral glucose tolerance test was performed in people without glucose-lowering medications.

**Results:**

The age-standardised prevalence of LVH was 9% (n=875) according to any of the three ECG criteria. Individuals with LVH were older, more likely to have hypertension, diabetes, known cardiovascular disease (CVD) and elevated levels of cholesterol and creatinine. Further, they were more likely to be of African descent (Creole) and have lower educational level. In a multivariable model, Creole (OR (95% CI)) (1.56 (1.33 to 1.83)), low educational level (1.49 (1.28 to 1.75)), hypertension (3.01 (2.55 to 3.56)), known CVD (1.42 (1.11 to 1.83)) and elevated creatinine (1.08 (1.03 to 1.14)) remained associated with LVH. Individuals with non-treated or uncontrolled hypertension had a higher risk for LVH (3.09 (95% CI 2.57 to 3.71) and 4.07 (95% CI 3.29 to 5.05), respectively), than individuals with well controlled hypertension or normotension.

**Conclusion:**

LVH occurs more frequently in individuals with hypertension, as well as in individuals with African ancestry and/or low education level.

**WHAT IS ALREADY KNOWN ON THIS TOPIC**

- Left ventricular hypertrophy (LVH) is a common condition, especially among individuals with hypertension. Present LVH is associated with a risk for cardiovascular disease.

**WHAT THIS STUDY ADDS**

- In a multiethnic community of mainly Asian and African descendants with a rapid socioeconomic growth, this study highlights the importance of well-regulated blood pressure, and the increased risk ethnicity and low socioeconomic status can mean for subgroups of the population.

**HOW THIS STUDY MIGHT AFFECT RESEARCH, PRACTICE OR POLICY**

- These results can help identify individuals at risk for LVH and assist policy-makers how and where to aim additional preventive measures.

## Introduction

Left ventricular hypertrophy (LVH) is defined as the thickening of the myocardium and leads to an increased risk of cardiovascular disease (CVD).^1^ The global prevalence of LVH is estimated to be 10%–20% in the adult population, whereas the prevalence is higher in individuals with hypertension. Approximately 40% of individuals with hypertension also have LVH.^2^ In hypertensive individuals with LVH, the risk for CVD is threefold higher.^3^ Older age, obesity and ethnic origin have also been found to be associated with LVH.

Cardiac resonance imaging is the most accurate method for detecting LVH but has a limited accessibility due to expensive and stationary equipment. Echocardiography (ECHO) is a more common method which has a higher sensitivity compared with a standard 12-lead ECG.^4^ However, as the ECHO method requires special training and long experience, which makes the ECG the most widely used screening method, especially in low-income and middle-income countries.^5^

The ECG records the electrical activity generated from the heart’s muscle cells, and the thickened heart muscle in LVH generates increased electrical activity. Consequently, the registered ECG complexes will have elevated amplitudes, which are classified based on established criteria.^6^

Mauritius has transitioned from a fishing and agricultural society (low-income country) to an upper middle-income country with a modern lifestyle and in July 2020 was considered a high-income country. However, due to COVID-10 pandemic, the raiting changed back to upper middle-income country in 2021.^7^ In parallel, a shift to increased prevalence of diabetes and hypertension has been observed.^8 9^ The Mauritian population is unique as its ethnicities represent a major proportion, of the global population including Asia and Africa.

To our knowledge, there are no contemporary studies presenting data on the prevalence of LVH and its determinates in a multiethic population including a large proportion of subjects with African ethnicity (creole).

The aim of this study was to determine the prevalence of LVH and associated factors in a multiethnic population from Mauritius.

## Methods

### Study design and individuals

The data were collected in Mauritius, a subtropical island located in the Indian Ocean with 1.3 million inhabitants. The resident population is composed of several ethnic groups; 68% Indo-Mauritian, 27% Creole (african ancestry), 3% Sino-Mauritian and 2% other ethnicities.

Two cross-sectional, population-based surveys were conducted in 2009 and in 2015 to estimate the prevalence of diabetes, CVD, hypertension and related risk factors. As previously described, several study sites were randomly selected, and one adult in every third household was randomly selected from these sites.^10^ To include adequate numbers of Sino-Mauritians, individuals from Chinatown in the capital city of Port Louis, were purposely selected.

**Figure 1:**
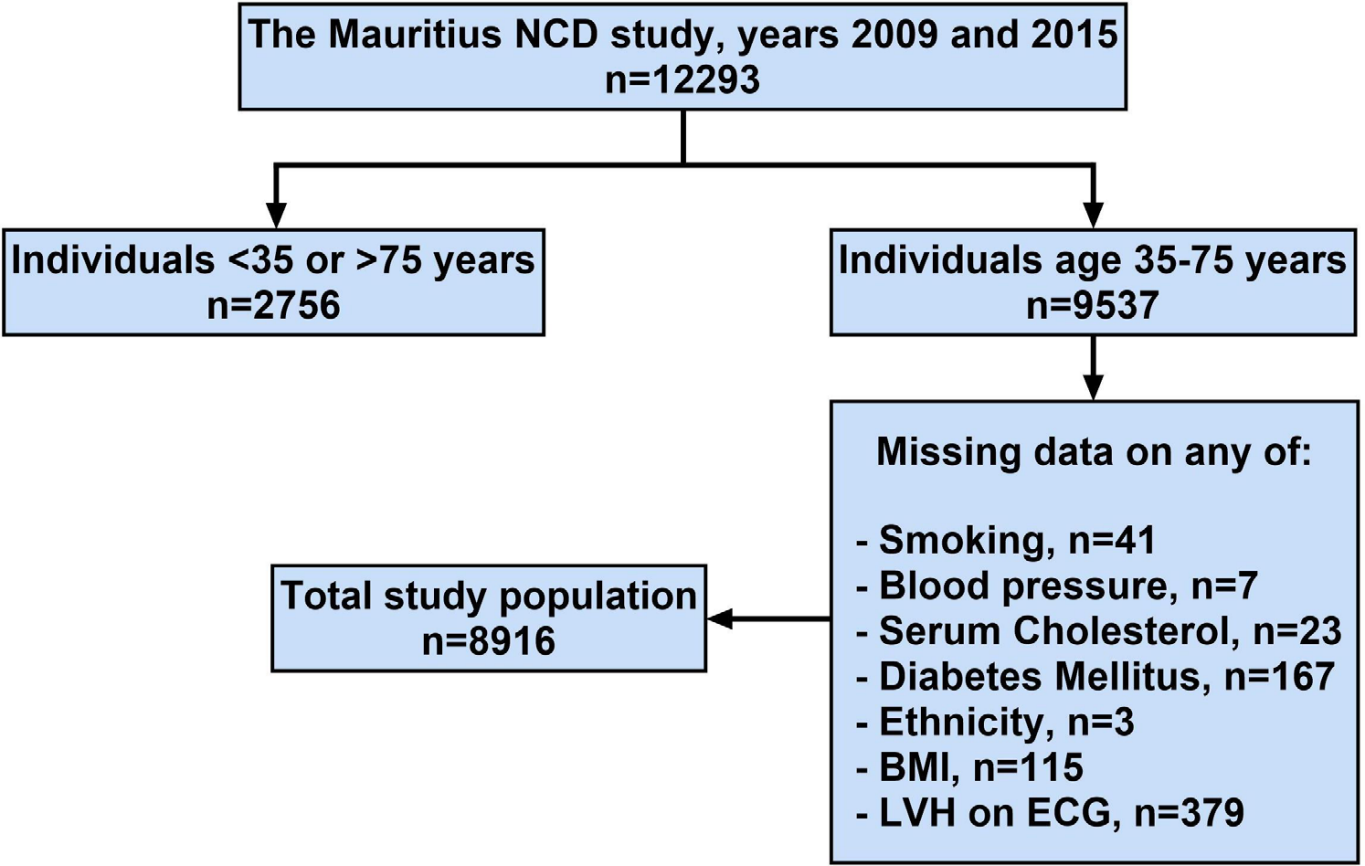
Flow chart describing the selection of study participants. BMI, body mass index; LVH, left ventricular hypertrophy; NCD, noncommunicable disease.

A total of 9537 unique individuals aged 35–74 years participated in the two surveys. Of these, 8916 individuals had a registered ECG and complete data on socioeconomic and cardiovascular risk factors. Numbers and reason for missingness are shown in figure 1.

The survey methods have been described previously.^8 11^ Briefly, participants were interviewed about ethnicity, smoking status, educational level (none or 1–6 years, 7–12 years or tertiary) and presence of CVD (ie, previous stroke, angina, heart attack, coronary bypass and angioplasty). Ethnicity was self-reported as Indo-Mauritian, Creole and Sino-Mauritian. Data on the awareness of diseases such as diabetes and hypertension and their treatment were obtained. Blood pressure was measured using automatic blood pressure monitors (Omron Digital Auto Blood Pressure Monitor SEM-1 in 2009 and Omron M7 in 2015 (Omron medical, Kyoto Japan)). Blood pressure was measured in the sitting position after 5 min of rest. Blood pressure was measured three times and the closest two measurements were averaged to provide the final blood pressure. Hypertension was defined as systolic blood pressure ≥140 mm Hg and/or diastolic blood pressure ≥90 mm Hg, or the use of antihypertensive drug treatment.^12^ Pulse pressure was obtained by subtracting diastolic blood pressure from systolic blood pressure. Blood pressure status was defined as: (1) normal blood pressure and no antihypertensive treatment, (2) normal blood pressure and antihypertensive treatment, (3) high blood pressure and no antihypertensive treatment and (4) high blood pressure and antihypertensive treatment. Anthropometric measurements (weight, height and waist and hip circumferences) were obtained.^10^

A venous blood sample was collected after an overnight fast. Serum lipids (total cholesterol, high-density lipoprotein (HDL), triglycerides), fasting and 2-hour plasma glucose and creatinine were analysed in a central laboratory. All individuals except those taking glucose-lowering drugs performed a 2-hour 75 g oral glucose tolerance test. Diabetes was defined as fasting plasma glucose ≥7.0 mmol/L and/or 2-hour postchallenge plasma glucose ≥11.1 mmol/L and/or using glucose-lowering drugs (oral medications or insulin) according to 2006 WHO recommendations.^13^

### ECG

A resting 12-lead ECG was recorded. Exclusion criteria were ongoing pregnancy or age below 35 years or above 75 years. LVH was defined according to any of three criteria: Sokolow-Lyon: SV1+ (RV5 or RV6)>35 mm (3.5 mV); Cornell voltage: SV3+RaVL>28 mm (2.8 mV) in men and SV3+RaVL>20 mm (2.0 mV) in women and Cornell product: (SV3+RaVL (+ 8 in women))×QRS duration >2.440 ms.^6^ All ECGs were coded by two independent coders, blinded to individuals’ medical history. Any differences in coding were reviewed and resolved by a consensus decision.

### Statistical analysis

Descriptive statistics were used to provide an overview of the study population. Prevalence data were age-standardised and sex standardised to the 2008 Mauritian population. To determine the significance between groups, Student’s t-test and χ^2^ tests were used as appropriate.

Survey-specific and sex-specific z-scores were calculated for fasting and 2-hour glucose, cholesterol and creatinine.

Univariable and multivariable logistic regression analyses were carried out to evaluate associations with LVH. Three multivariable models were used. In model 1, background and cardiovascular factors were included (age, sex, survey year, hypertension, diabetes, creatinine, smoking, cholesterol and known CVD). In model 2, ethnicity and educational levels were added, and in model 3, hypertension (Y/N) was replaced with hypertension control (four levels, see above). Systolic, diastolic blood pressure, pulse pressure and blood pressure control replaced hypertension, and fasting and 2-hour glucose levels replaced diabetes status in separate models. Interactions were tested by stratified analyses based on ethnicity, educational level, presence of CVD and sex. CI was set to 95% and a p<0.05 was considered statistically significant. SPSS V.27 (IBM) was used for all statistical calculations.

### Patient and public involvement

Patients were not involved in setting the research question, the outcome measures or the design or implementation of the study reported in this paper.

## Results

### Prevalence of LVH

Altogether, 8916 individuals were included in this study, and 4390 participated in 2009 and 4526 in 2015. The mean age was 51.9 years (95% CI 51.6 to 52.2) in 2009 and 55.5 years (95% CI 55.2 to 55.8) in 2015. Altogether 875 individuals had LVH according to any of the three criteria, which gives an age-standardised prevalence of 9.0% (95% CI 8.4% to 9.6%). The age-standardised prevalence did not differ between 2009 and 2015 (8.5% (95% CI 7.7% to 9.3%) and 9.6% (95% CI 8.8% to 10.5%), respectively), or between men and women (9.4% (95% CI 8.5% to 10.3%) and 8.6% (95% CI 7.8% to 9.4%), respectively). The age-standardised prevalence was higher in Creoles (13.5% (95% CI 11.9% to 15.0%)) than in Indo-Mauritians (7.9% (95% CI 7.3% to 8.6%)) and Sino-Mauritians (7.7% (95% CI 4.8% to 10.6%)) (table 1).

**Table 1:** Population characteristics

	No LVH	LVH	P value
	n=8041	n=875	
Age (years)	53.4 (53.1–53.6)	56.6 (55.9–57.3)	<0.001
Female sex (%)	54.7 (53.6–55.8)	56.8 (53.5–60.1)	0.24
Systolic BP (mm Hg)	131.2 (130.7–131.7)	151.1 (149.3–152.9)	<0.001
Diastolic BP (mm Hg)	80.3 (80.1–80.6)	87.5 (86.6–88.4)	<0.001
Pulse pressure (mm Hg)	50.9 (50.6–51.3)	63.6 (62.2–65)	<0.001
Hypertension (%)	44.1 (43–45.2)	72.3 (69.4–75.3)	<0.001
Fasting glucose (mmol/L)	6.8 (6.7–6.8)	7.0 (6.9–7.2)	0.006
2-hour glucose (mmol/L)	7.7 (7.7–7.8)	8.1 (7.8–8.3)	0.02
Diabetes (%)	33.6 (32.6–34.7)	37.5 (34.3–40.7)	0.03
Creatinine (µmol/L)	84.2 (83.7–84.7)	89.8 (86.2–93.4)	0.003
BMI (kg/m^2^)	26.1 (26–26.2)	26.2 (25.9–26.5)	0.52
Waist (cm)	87.4 (87.2–87.7)	87.6 (86.8–88.3)	0.73
Current smoker (%)	16.7 (15.9–17.5)	17.9 (15.4–20.5)	0.34
Cholesterol (mmol/L)	5.2 (5.2–5.2)	5.3 (5.2–5.4)	0.02
Known CVD (%)	5.8 (5.2–6.3)	10.6 (8.5–12.6)	<0.001
Ethnicity n (%)			<0.001
Indo Mauritian	6067 (76)	584 (67)	
Creole	1653 (21)	272 (31)	
Sino Mauritian	313 (4)	19 (2)	
Educational level n (%)			<0.001
None or primary 1–6 years	4009 (50)	566 (65)	
Secondary 1–6 years	3548 (44)	280 (32)	
Tertiary	464 (6)	27 (3)	
BP control (%)			<0.001
No treatment and good level	56	28	
Treatment and good level	8	7	
No treatment and high BP	24	38	
Treatment and high BP	12	27	

Hypertension=systolic BP≥140 and/or diastolic BP≥90 and/or on treatment, and pulse pressure=systolic BP−diastolic BP.

Values shown are means and proportions with (95% CIs) and numbers.

Significance tested with Student’s t-test or χ^2^ tests when appropriate.

BMI, body mass index; BP, blood pressure; CVD, cardiovascular disease; LVH, left ventricular hypertrophy.

The prevalence of LVH decreased with higher level of educational attainment. The prevalence of LVH in those with 6 or fewer years of education was 11.1% (95% CI 10.2% to 12.0%)) compared with individuals with 7–12 years of education (7.2% (95% CI 6.4% to 8.1%)) or a tertiary level of education (5.1% (95% CI 3.2% to 7.1%)) (see also table 1).

**Figure 2:**
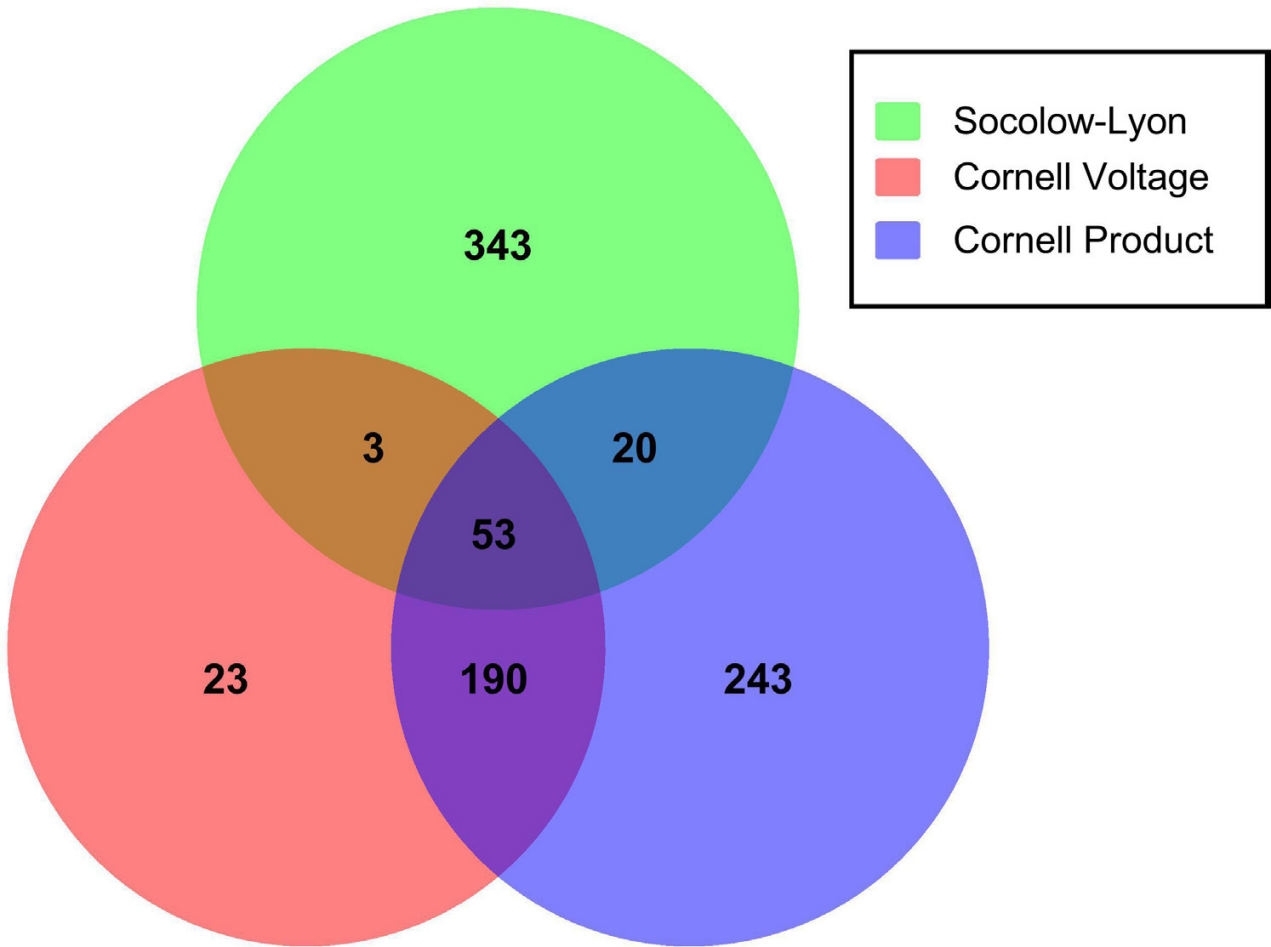
Venn diagram illustrating findings of left ventricular hypertrophy (LVH) based on detection criteria Sokolow-Lyon (SL), Cornell product (CP) and Cornell voltage (CV). The distribution of LVH according to SL (n=343+3+53+20; 47.9%), CV (n=23+3+53+190; 30.7%) and CP (n=243+20+53+190; 57.8%). Overlapping areas: 18.1% of those detected with SL were also detected with and CV and/or CP, 91.4% of those detected with CV were also detected with and SL and/or CP Cornell voltage, and 52.0% of those detected with CP were also detected with and SL and/or CV.

Among the 875 individuals with LVH, a total of 419 (48%) fulfilled the Sokolow-Lyon criteria, 269 (31%) individuals fulfilled the Cornell voltage, and 506 (58%) fulfilled the Cornell product. The criteria overlapped, and 18% of those detected by Sokolow-Lyon were also detected by Cornell voltage and or Cornell product, 91% of those detected by Cornell voltage were also detected by Sokolow-Lyon and Cornell product, and 52% of those detected by Cornell product were also detected by Sokolow-Lyon and Cornell voltage (figure 2).

### Associated factors

Individuals with LVH were older and had higher systolic and diastolic blood pressure and were more likely to have hypertension (table 1). Furthermore, they were more likely to have non-treated or treated but uncontrolled hypertension. The prevalence of LVH was among individuals with hypertension 14.3% (95% CI 13.3% to 15.4%) compared with 5.2% (95% CI 4.5% to 5.8%) among those without hypertension. Their glucose levels were higher, and they were more likely to have diabetes. Their creatinine and cholesterol levels were higher.

In the univariable logistic regression analysis, age, survey year, hypertension, high systolic and diastolic blood pressure, high pulse pressure, high fasting, and 2-hour glucose levels, diabetes, high creatinine, and cholesterol levels, known CVD, Creole ethnicity and lower educational level were associated with the presence of LVH. Anthropometric measures and smoking did not associate with LVH (table 2).

**Table 2:** Univariable and multivariable logistic regression

	Univariable	Model 1	Model 2	Model 3
LVH/no LVH (n)	875/8041			
Age (year)	1.03 (1.03–1.04)	1.01 (1.00–1.02)	1.01 (1.00–1.02)	1.01 (1.00–1.02)
Sex (female vs men)	1.09 (0.95–1.25)	1.23 (1.05–1.45)	1.08 (0.92–1.28)	1.11 (0.94–1.31)
Survey (2015 vs 2009)	1.24 (1.08–1.43)	1.34 (1.16–1.55)	1.39 (1.19–1.61)	1.46 (1.26–1.70)
Systolic BP (mm Hg)	1.03 (1.03–1.03)			
Diastolic BP (mm Hg)	1.05 (1.04–1.05)			
Pulse pressure (mm Hg)	1.04 (1.03–1.04)			
Hypertension (Y/N)	3.31 (2.84–3.87)	3.15 (2.67–3.72)	3.01 (2.55–3.56)	
Fasting glucose (Z-score)	1.09 (1.03–1.16)			
2 hour glucose (Z-score)	1.11 (1.03–1.19)			
Diabetes (Y/N)	1.18 (1.02–1.37)	0.81 (0.70–0.95)	0.81 (0.69–0.94)	0.80 (0.68–0.94)
Creatinine (Z-score)	1.14 (1.09–1.20)	1.09 (1.03–1.14)	1.08 (1.03–1.14)	1.07 (1.02–1.13)
BMI (kg/m^2^)	1.01 (0.99–1.02)			
Waist (cm)	1.00 (0.99–1.01)			
Smoking (Y/N)	1.09 (0.91–1.31)	1.35 (1.10–1.67)	1.19 (0.96–1.47)	1.21 (0.98–1.49)
Cholesterol (Z-score)	1.08 (1.01–1.16)	1.07 (1.00–1.14)	1.07 (0.99–1.14)	1.04 (0.97–1.12)
Known CVD	1.94 (1.53–2.45)	1.47 (1.14–1.88)	1.42 (1.11–1.83)	1.47 (1.14–1.90)
Ethnicity				
Indo-Mauritian	1.00		1.00	1.00
Creole	1.71 (1.47–2.00)		1.56 (1.33–1.83)	1.54 (1.31–1.81)
Sino-Mauritian	0.63 (0.39–1.01)		0.71 (0.44–1.15)	0.75 (0.46–1.22)
Educational level				
None or primary 1–6 years	1.00		1.00	1.00
Secondary 1–6 years	0.56 (0.48–0.65)		0.68 (0.58–0.80)	0.69 (0.58–0.81)
Tertiary	0.41 (0.28–0.61)		0.59 (0.39–0.88)	0.61 (0.40–0.92)
BP control				
No treatment and good level	1.00			1.00
Treatment and good level	1.73 (1.30–2.32)			1.54 (1.14–2.09)
No treatment and high BP	3.22 (2.70–3.83)			3.09 (2.57–3.71)
Treatment and high BP	4.60 (3.80–5.57)			4.07 (3.29–5.05)

Values shown are ORs and (95% CIs).

BMI, body mass index; BP, blood pressure; CVD, cardiovascular disease; Y/N, yes/no.

After adjustment according to model 1, hypertension, creatinine and known CVD remained significantly associated with LVH (table 2). Further adjustments for educational level and ethnicity did not attenuate these associations (model 2). In model 2, Creole ethnicity remained associated with increased risk for LVH whereas higher educational level associated with lower risk for LVH.

Replacing hypertension with systolic, diastolic or pulse pressure, gave similar results (data not shown). Similarly, replacing diabetes status with fasting and 2-hour glucose levels did not add any extra information (diabetes and glucose levels did not associate with LVH in the adjusted model (not shown)).

All levels of blood pressure control were associated with the presence of LVH compared with normotension, (normal pressure and treatment (1.54 (95% CI 1.14 to 2.09)), high pressure and no treatment (3.09 (95% CI 2.57 to 3.71)) and high pressure and treatment (4.07 (95% CI 3.29 to 5.05)) (model 3).

Stratified analyses were done based on ethnicity, educational level, sex and presence of CVD (table 3).

**Table 3:** Multivariable logistic regression stratified for ethnicity and sex

	Indo-Mauritian	Creole	Sino-Mauritian	Men	Women
LVH/no LVH, n	584/6067	272/1653	19/313	378/3641	497/4400
Age (per year)	1.02 (1.01–1.03)	1.00 (0.99–1.02)	0.96 (0.91–1.01)	0.99 (0.98–1.00)	1.03 (1.02–1.04)
Sex (female vs men)	1.27 (1.03–1.57)	0.86 (0.64–1.14)	0.43 (0.14–1.32)		
Survey (2015 vs 2009)	1.38 (1.15–1.65)	1.37 (1.04–1.79)	1.19 (0.44–3.24)	1.51 (1.21–1.89)	1.29 (1.06–1.58)
Hypertension (Y/N)	2.86 (2.33–3.49)	3.35 (2.44–4.60)	2.84 (0.94–8.59)	2.46 (1.93–3.13)	3.54 (2.80–4.47)
Diabetes (Y/N)	0.79 (0.66–0.96)	0.81 (0.60–1.08)	1.55 (0.51–4.69)	0.68 (0.53–0.87)	0.91 (0.74–1.12)
Creatinine (Z-score)	1.08 (1.01–1.15)	1.08 (0.98–1.18)	1.33 (0.81–2.21)	1.13 (1.05–1.21)	1.03 (0.95–1.10)
Smoking (Y/N)	1.42 (1.08–1.88)	0.88 (0.63–1.25)	1.96 (0.46–8.36)	1.18 (0.94–1.48)	0.93 (0.50–1.72)
Cholesterol (Z-score)	1.12 (1.03–1.21)	0.95 (0.83–1.09)	1.14 (0.70–1.86)	1.04 (0.93–1.15)	1.05 (0.95–1.15)
Known CVD	1.44 (1.07–1.93)	1.44 (0.90–2.31)	–	1.25 (0.86–1.82)	1.67 (1.19–2.35)
Educational level					
None or primary 1–6 years	1.00	1.00	1.00	1.00	1.00
Secondary 1–6 years	0.65 (0.53–0.79)	0.82 (0.62–1.08)	0.71 (0.16–3.04)	0.69 (0.55–0.87)	0.72 (0.57–0.91)
Tertiary	0.63 (0.39–1.01)	0.23 (0.06–0.97)	1.26 (0.23–6.79)	0.60 (0.37–0.97)	0.56 (0.24–1.29)
Ethnicity					
Indo-Mauritian				1.00	1.00
Creole				1.82 (1.44–2.31)	1.42 (1.14–1.78)
Sino-Mauritian				1.21 (0.67–2.18)	0.32 (0.13–0.79)

Values shown are ORs and (95% CIs).

CVD, cardiovascular disease; LVH, left ventricular hypertrophy; Y/N, yes/no.

In these analysis, higher educational level was associated with less risk for LVH in Indo-Mauritians, Creoles, men and women and participants without CVD. The point estimate was similar in Sino-Mauritians and in those with known CVD, although not significant. Creole ethnicity was associated with a higher risk for LVH in both men and women and in participants without CVD. The point estimate was similar in those with known CVD, although not significant (few individuals). Participants with Creole ethnicity had a higher risk for LVH irrespective of educational level (none or primary school 1.41 (95% CI 1.15 to 1.72), and secondary or tertiary school 1.89 (95% CI 1.45 to 2.46)).

## Discussion

In this study, we found that the presence of LVH associated strongly with the degree of blood pressure control. Furthermore, Creole ethnicity and educational level were important determinants of LVH, even after adjustment for cardiovascular risk factors.

The age-standardised prevalence of LVH in our study was 9%. In a review comparing 26 ECG-LVH studies with 40 444 individuals, the average prevalence of LVH was 18%. However, all individuals had hypertension, a well-known risk factor for developing LVH.^14^ Our study included both normotensive and hypertensive individuals which could explain a lower prevalence. Furthermore, the previously mentioned studies included several other criteria for detecting LVH, such as Minnesota codes, Romhilt-Estes, etc^14^ while we used Sokolow-Lyon, Cornell voltage and Cornell product. A cross-sectional population-based study from Thailand showed a lower prevalence (6%) of LVH. However, that study used only the Sokolow-Lyon and Cornell voltage criteria.^15^ In our study, the majority of individuals with LVH were detected with Cornell product. The Cornell criteria has in other studies been shown to detect a higher rate of LVH compared with the Sokolow-Lyon criteria.^14^

In this study, hypertension associated strongly with the presence of LVH, and the degree of blood pressure control was important, with the lowest risk for LVH seen in individuals with normal blood pressure, as well as those with achieved blood pressure control. This is in line with previous studies.^14^

We demonstrated that increasing age was associated with a higher prevalence of LVH which is consistent with previous studies.^16^ However, we did not find that sex was associated with LVH, despite other studies showing that there is a higher prevalence of LVH in men, probably due to higher prevalence of cardiovascular risk factors in men.^17 18^ Interestingly, our results showed that female sex associated with LVH in participants with known CVD, findings that should be interpreted cautiously due to small numbers.^16 19–21^

Hypertensive individuals of African ethnicity have also been shown to have a larger ventricular mass as compared with hypertensive people of other ethnicities.^19–22^ The relatively high prevalence in Creoles is similar to the prevalence reported in a Nigerian population (16.4%), although the authors only used the Sokolow-Lyon criteria.^23^ The prevalence of LVH among Indo-Mauritians (9%) was similar to the 6% prevalence found in a study from New Delhi in India.^24^ The Sino-Mauritian population in our study had a similar prevalence compared with Chinese (5%) and Thai (6%) populations.^15 25^

Individuals with lower education levels had a higher prevalence of LVH in Mauritius, even after adjustment for ethnicity and other risk factors, notably, irrespective of ethnicity, sex and presence of CVD. Historically, individuals with African ethnicity have had a lower level of education compared with other ethnic groups in Mauritius.^26^ In this study, we used the educational level as a proxy for socioeconomic status (SES). Previous studies have shown increased morbidity and mortality in individuals with lower SES compared with those with higher SES, and the individuals with lower SES were more likely to be hypertensive and had higher psychological stress, which could contribute to the development of LVH.^27 28^

Elevated body mass index (BMI) may cause a lower QRS-amplitude on ECGs.^17 18^ This is especially seen when using the Sokolow-Lyon criteria, which is the criterion with the lowest sensitivity (≈20%), but the highest specificity (>85%), compared with the Cornell voltage (sensitivity 42%, specificity 95%) and the Cornell product (sensitivity 51%, specificity of 95%).^6^ Obesity is thus an important confounding factor that can both affect the ECG amplitudes and the development of hypertension and diabetes.^29^ However, in our study, neither BMI nor waist circumference were related to the prevalence of LVH.

The finding that diabetes was a statistically significant predictor of LVH in univariable analysis, but no longer after multiple adjustments in multivariable analysis suggests that collinearity between the diabetes status and some other predictors of LVH are likely to exist. This issue will be evaluated in more detailed analyses of the data in the future and results will be reported separately.

### Strengths and limitations

The strength of our study is a large population-based cohort, structured standardised data collection and the usage of three well-established ECG criteria for detecting LVH. QRS-amplitude was manually measured by independent coders, and any discrepancies were solved by consensus. The large population-based study cohort on randomly selected individuals included the three major ethnicities in Mauritius. There are also limitations to this study: we only used ECG for the detection of LVH, which has a lower sensitivity compared with ECHO. However, ECG is still a common method for detecting LVH, especially in low-income and middle-income countries. We used three definitions of LVH on ECG, it is possible that including more definitions could have identified more individuals with LVH. Other limitations are that previous history of CVD, smoking habits and education level have been obtained by self-report. However, standardised methods of data collection and consistency over time should be emphasised. The individuals in the cohort from 2015 were significantly older than those in 2009 cohort; however, the analysis was adjusted for both age and survey year.

## Conclusion

In conclusion, LVH has a higher prevalence in individuals with hypertension, with Creole ethnicity and/or low educational level. This study shows the importance of adequate blood pressure control in relation to LVH. Further research is needed to elaborate the relation between diabetes and LVH and the mechanisms explaining the association between SES and CVD.

## Data Availability

Data are available on reasonable request. The data collected from the population-based surveys in Mauritius contain personal sensitive information and cannot be shared publicly. On reasonable request to the corresponding author, and after review by the Ministry of Health and Wellness, Mauritius, study protocols can be made available.
